# Identification of Potential miRNA Biomarkers to Detect Hydrocortisone Administration in Horses

**DOI:** 10.3390/ijms241914515

**Published:** 2023-09-25

**Authors:** Mio Kikuchi, Taichiro Ishige, Yohei Minamijima, Kei-ichi Hirota, Shun-ichi Nagata, Teruaki Tozaki, Hironaga Kakoi, Toshina Ishiguro-Oonuma, Keiichiro Kizaki

**Affiliations:** 1 Genetic Analysis Department, Laboratory of Racing Chemistry, 1731-2 Tsurutamachi, Utsunomiya 320-0851, Tochigi, Japan; m-kikuchi@lrc.or.jp (M.K.);; 2 Graduate School of Veterinary Sciences, Iwate University, 3-18-8 Ueda, Morioka 020-8550, Iwate, Japan; 3 Drug Analysis Department, Laboratory of Racing Chemistry, 1731-2 Tsurutamachi, Utsunomiya 320-0851, Tochigi, Japan

**Keywords:** horse, microRNA, hydrocortisone, doping, biomarkers

## Abstract

Circulating microRNAs (miRNAs) are stable in bodily fluids and are potential biomarkers of various diseases and physiological states. Although several studies have been conducted on humans to detect drug doping by miRNAs, research on drugs and miRNAs in horses is limited. In this study, circulating miRNAs in horses after hydrocortisone administration were profiled and variations in miRNAs affected by hydrocortisone administration during endogenous hydrocortisone elevation were examined. The miRNAs were extracted from thoroughbred horse plasma before and after hydrocortisone administration and subjected to small RNA sequencing and reverse transcription quantitative PCR (RT-qPCR). RT-qPCR validation was performed for the 20 miRNAs that were most affected by hydrocortisone administration. The effects of elevated endogenous hydrocortisone levels due to exercise and adrenocorticotropic hormone administration were also confirmed. The validation results showed that approximately half of the miRNAs showed the same significant differences as those obtained using small RNA sequencing. Among the twenty miRNAs, two novel miRNAs and miR-133a were found to vary differently between exogenous hydrocortisone administration and endogenous hydrocortisone elevation. This study provides basic knowledge regarding the circulating miRNA profile of horses after hydrocortisone administration and identifies three miRNAs that could potentially be used as biomarkers to detect hydrocortisone administration.

## 1. Introduction

MicroRNAs (miRNAs) are small, single-stranded, non-coding RNAs that bind to mRNAs with complementary sequences and inhibit translation, thereby repressing the expression of target proteins [1]. Some mature miRNAs synthesized intracellularly are released from the cell and are either bound to proteins and lipids or encapsulated in exosomes [2]. The miRNAs are released extracellularly into the blood in a stable state, unaffected by RNA-degrading enzymes [3]. Circulating miRNAs in bodily fluids regulate various in vivo phenomena, including cell proliferation, apoptosis, development, immune modulation, and hormone secretion [4]. They are considered valuable biomarkers for various diseases and physiological phenomena, such as cancer, Alzheimer’s disease, rheumatoid arthritis, obesity, preeclampsia, and pregnancy [3,5,6,7].

In humans, miRNA research for anti-doping purposes in athletes is ongoing and several miRNAs have been reported to be altered by autologous blood transfusion [8], the administration of testosterone [9], erythropoiesis-stimulating agents [10], growth hormones [11], and dexamethasone [12]. Drug testing for doping control has also been conducted in racehorses and the profile of eca-miR-144 levels in horse plasma treated with erythropoiesis-stimulating factor products has been reported with reference to previous human studies [13]. However, there are few reports on circulating miRNA levels after drug administration in horses [13] and further studies are needed.

Hydrocortisone is a steroid hormone secreted by the adrenal cortex under the control of adrenocorticotropic hormone (ACTH) from the anterior pituitary gland. Its secretion is altered by various stresses and the circadian rhythm [14,15,16]. It is also used in preparations for its anti-inflammatory properties and is commonly used in equine clinical settings [17]. Hydrocortisone and other glucocorticoid preparations are prohibited substances in horse racing and equestrian sports and are subject to drug testing for doping control. Direct analytical LC-MS/MS is the most commonly used method for detecting hydrocortisone preparations in doping controls; however, this method cannot determine whether hydrocortisone is exogenous or endogenous. Thus, novel indirect approaches are needed to address such issues. 

To validate the applicability of miRNAs for drug testing in hydrocortisone detection, it is essential to gather data on circulating miRNA levels following hydrocortisone administration. However, no studies have investigated the effects of hydrocortisone on circulating miRNAs in horses. Therefore, in this study, hydrocortisone was administered to thoroughbred horses and plasma miRNA profiling was performed using small RNA sequencing (small RNA-seq). The miRNAs most affected by hydrocortisone administration were selected as candidate miRNAs for drug testing. In addition, the levels of the candidate miRNAs selected from the RNA-seq results were examined when endogenous hydrocortisone synthesis was increased to verify whether the miRNAs could distinguish between exogenous and endogenous hydrocortisone.

## 2. Results

### 2.1. Analysis of Circulating miRNA Profiles in Horses after Hydrocortisone Administration Using RNA-seq

**Figure 1:**
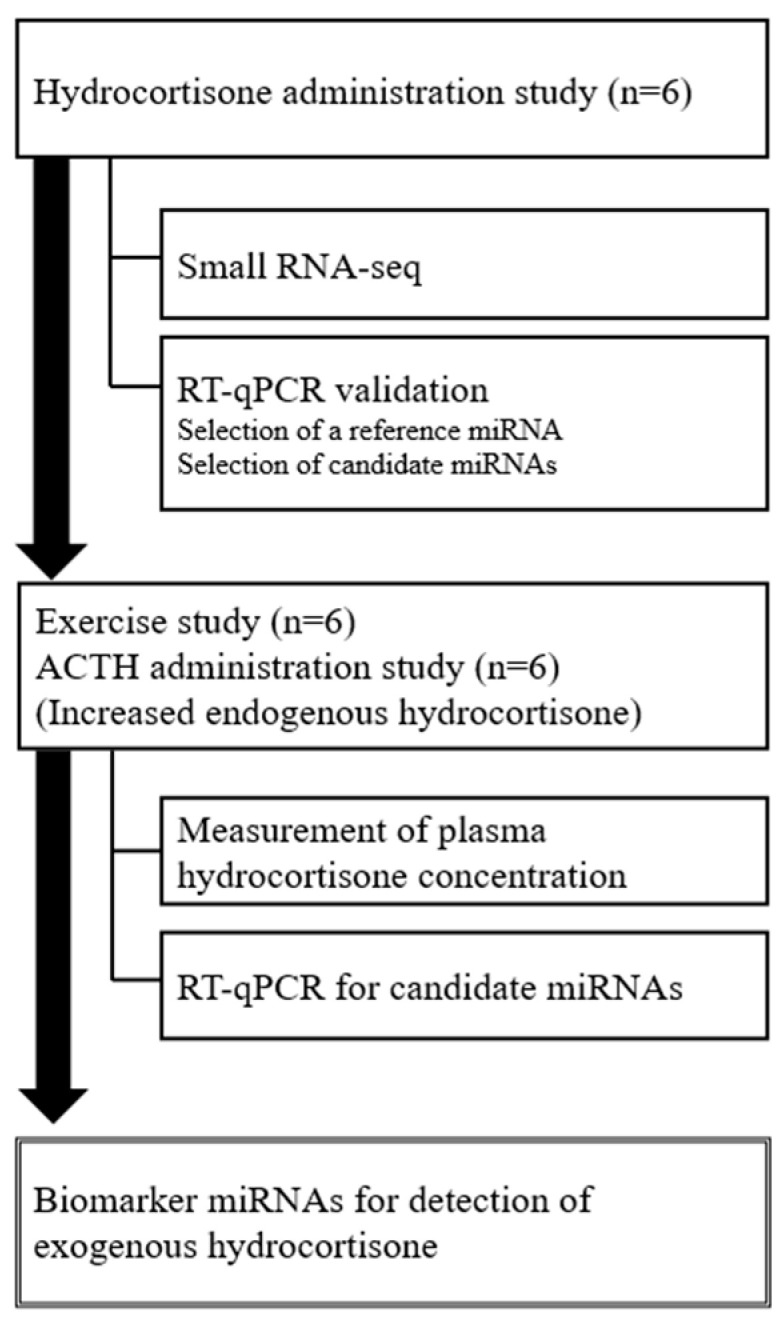
Study design. Experimental protocol for the identification of miRNA biomarkers to detect hydrocortisone administration.

The study design is shown in Figure 1. 

The analysis of plasma samples obtained from the hydrocortisone administration experiments using small RNA-seq yielded approximately 12–18 million reads per sample, approximately 20–40% of which were mapped to the genome (EquCab3) (Table 1). A total of 600–700 miRNAs, both novel and known miRNAs, were detected in each sample.

**Figure 2:**
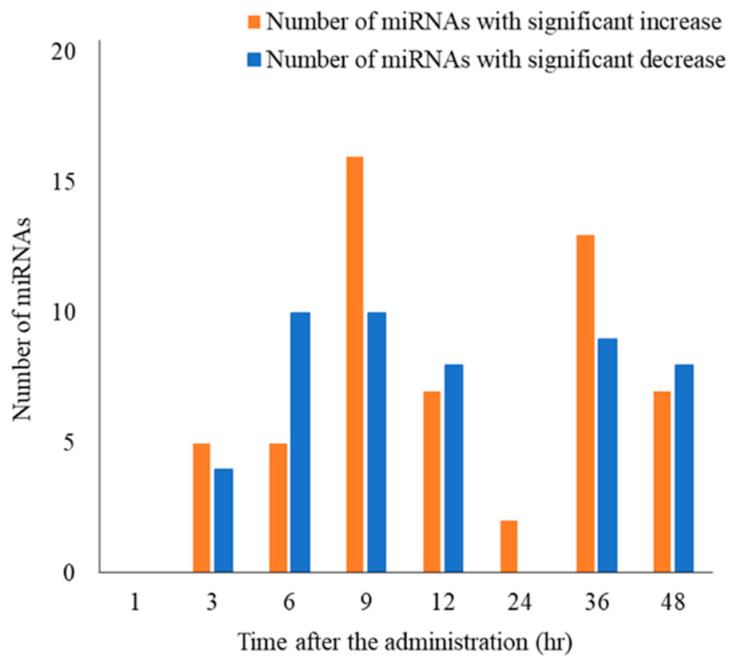
The number of differentially expressed circulating miRNAs after hydrocortisone administration. Differentially expressed circulating miRNAs were summarized by the direction of changes in increase or decrease after hydrocortisone administration compared with time-matched controls. Fold-changes were calculated as log2 (after administration/time-matched control), indicating the significant changes of |log2 fold-change| ≥ 1 after hydrocortisone administration at each post-dose time point by small RNA-seq (adjusted *p*-value: padj < 0.05).

**Figure 3:**
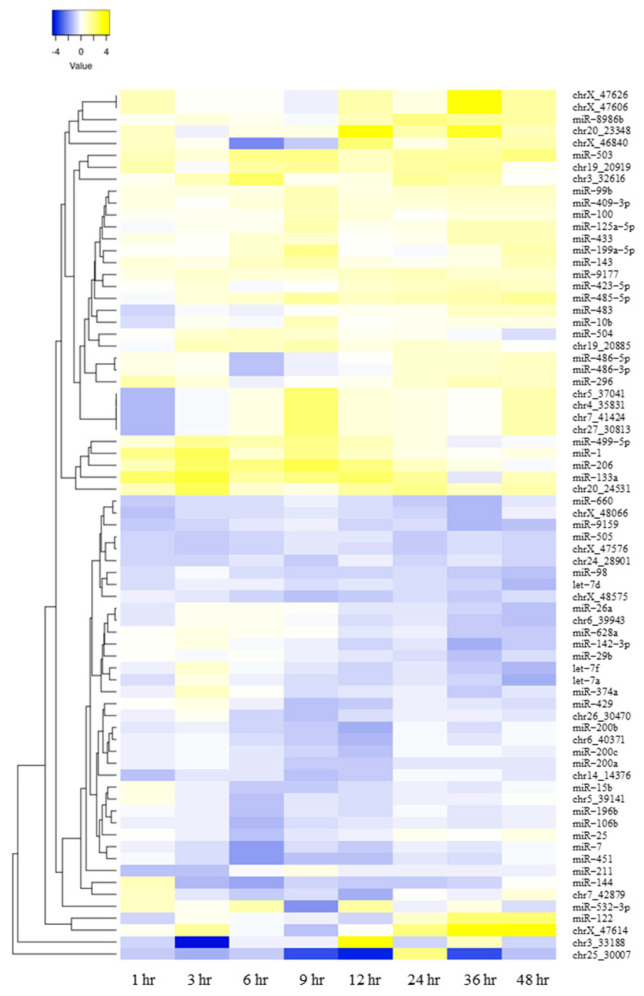
Hierarchical clustering heat map of the expression of differentially expressed circulating miRNAs after hydrocortisone administration. The log2 fold-change value of 73 miRNAs with a significant variation of |log2 fold-change| ≥ 1 by small RNA-seq was indicated in the dendrogram of the hierarchical clustering heat map. Fold changes were calculated as log2 (after administration/time-matched control). The miRNAs in yellow and violet indicate high and low expression, respectively. The log2 fold-change of chr3_33188 was out of range at −11.37 at 3 h and 8.66 at 12 h after administration.

The number of miRNAs with more than a significant two-fold (|log2 fold-change| ≥ 1) difference after hydrocortisone administration is shown in Figure 2. No miRNAs showed significant differences at 1 h after administration; however, at each time point from 3 to 48 h after administration, significant differences in |log2 fold-change| ≥ 1 were observed for 2–26 miRNAs, including both novel and known miRNAs. The log2 fold-change values of the 73 miRNAs with significant differences in |log2 fold-change| ≥ 1 are shown in a heat map (Figure 3). Compared to the time-matched controls, approximately half of the miRNAs showed an increasing trend; whereas, the other half showed a decreasing trend.

### 2.2. Validation of miRNA Levels after Hydrocortisone Administration Using Reverse Transcription-Quantitative PCR (RT-qPCR)

The results of the reference miRNA selection for RT-qPCR are shown in Table 2. The miRNAs evaluated were cel-miR-39 (a spike-in control), miR-191a, let-7g, miR-128, and miR-146a, which were stable and unaffected by hydrocortisone administration. All miRNAs had a standard deviation (SD) of <1 and miR-146a had the highest correlation coefficient.

Among the 73 miRNAs with significant differences in |log2 fold-change| ≥ 1 in small RNA-seq, the top 10 miRNAs with the highest |log2 fold-change| values for each increase and decrease were selected and RT-qPCR was performed. The results are presented in Table 3 and Supplementary Figure S1. As with small RNA-seq, nine miRNAs showed significant differences (*p* < 0.05) and there were two miRNAs with *p* < 0.1 (Table 3). No miRNAs showed significant changes opposite to the small RNA-seq results. The |log2 fold-change| values obtained using RT-qPCR were lower than those obtained using small RNA-seq.

### 2.3. Measurement of Plasma Hydrocortisone Concentration after Exercise and ACTH Administration

**Figure 4:**
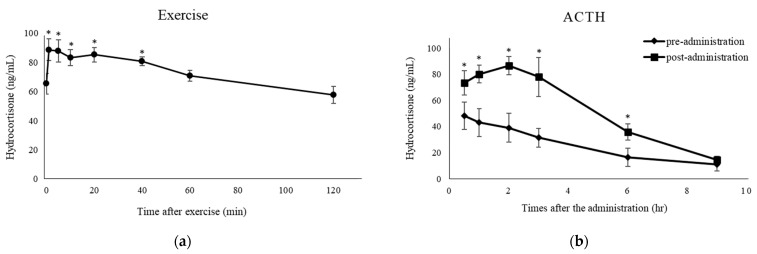
Endogenous hydrocortisone concentrations in plasma after exercise or ACTH administration. (**a**) Plasma hydrocortisone concentrations after exercise on a treadmill. The hydrocortisone concentrations were compared with the values before exercise. (**b**) Plasma hydrocortisone concentrations after administration of ACTH preparation (1.0 µg/kg body weight; closed diamond-shaped). Hydrocortisone concentrations were compared to the pre-administration concentrations (time-matched control; closed square). Data are shown as mean ± SD. *: Significantly higher than no treatment (*p* < 0.05).

The mean plasma concentrations of hydrocortisone in the exercise and ACTH administration groups are shown in Figure 4. Significant increases in hydrocortisone concentrations were observed from 1–40 min after exercise compared with immediately before exercise. Significant increases in hydrocortisone concentrations were also observed from 0.5–6 h after ACTH administration compared to the time-matched controls.

### 2.4. Analysis of miRNA Levels after Exercise and ACTH Administration Using RT-qPCR

The 20 most affected miRNAs after hydrocortisone administration were verified to change during endogenous hydrocortisone elevation in the plasma after exercise or ACTH administration. One of the six horses used in the exercise and ACTH administration studies was excluded from the RT-qPCR analysis in the exercise study because of low plasma miRNA abundance and a lack of amplification of several miRNAs during the exercise study period.

**Figure 5:**
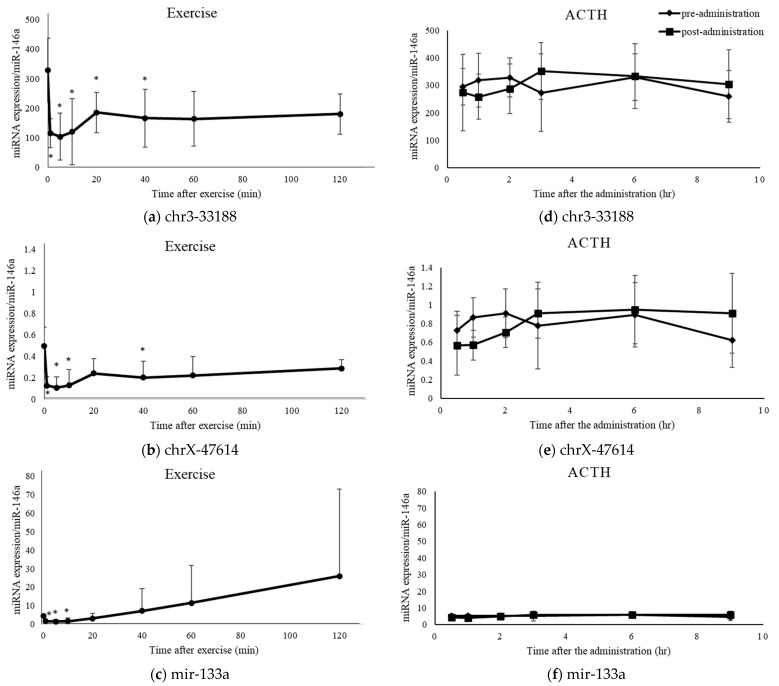
Levels of three miRNAs (chr3-33188, chrX-47614, and miR-133a) after exercise or ACTH administration. The miRNA levels of (**a**) chr3-33188, (**b**) chrX-47614, and (**c**) miR-133a after exercise on a treadmill. The miRNA levels were compared with the values before exercise. The miRNA levels of (**d**) chr3-33188, (**e**) chrX-47614, and (**f**) miR-133a after the administration of ACTH preparation (1.0 µg/kg BW; closed diamond-shaped). The miRNA levels were compared to the pre-administration levels (time-matched control; closed square). Data are shown as mean ± SD. *: Significant differences between pre- and post-exercise or post-administration of ACTH (*p* < 0.05).

Among the miRNAs for which RT-qPCR was performed, the novel miRNAs chr3-33188 and chrX-47614 and the known miRNA miR-133a were down-regulated after exercise and not changed after ACTH administration (Figure 5). These three miRNAs were up-regulated after hydrocortisone administration and, therefore, showed different variations during endogenous hydrocortisone elevation compared to those during exogenous hydrocortisone elevation. Variations in other miRNAs during endogenous hydrocortisone elevation in the plasma were similar to those observed during hydrocortisone administration (Supplementary Figure S2).

## 3. Discussion

This is the first report to analyze circulating miRNA profiles in horses after hydrocortisone administration. The most affected miRNAs after hydrocortisone administration were also verified these variations during exercise and ACTH treatment when endogenous hydrocortisone levels increased. As a result, different variations were observed for two novel miRNAs and miR-133a following exogenous hydrocortisone treatment and an endogenous hydrocortisone increase.

Similar to other mammals, the circadian rhythm of endogenous hydrocortisone has been reported in horses [18,19]. In resting horses, hydrocortisone levels are known to slowly increase from dawn, peak in the morning, and then gradually decrease, reaching their lowest levels in the evening and at night. This same circadian rhythm of endogenous hydrocortisone levels was also observed in our previous study [20]. In this study, control samples were collected on separate days prior to the administration of hydrocortisone and ACTH. This approach necessitated a longer sampling period to ensure that the control samples accurately represented the pre-administration baseline. Therefore, accurate miRNA profiles reflecting the circadian rhythm of endogenous hydrocortisone were achieved. In addition, compared with previous studies investigating drug–miRNA associations in humans and horses [9,12,13], more time points were set for sampling after drug administration in this study, allowing for a more detailed assessment of miRNA variation.

Approximately 700 equine miRNAs are registered in miRBase (http://www.mirbase.org (accessed on 27 July 2023)), which is a small number of miRNAs compared to other mammals, such as humans, mice, and cattle. Therefore, in the present study, small RNA-seq was performed for miRNA analysis. Small RNA-seq analysis obtains sequence information from reads using next-generation sequencing and can detect novel miRNAs that are not registered in the database [4]. In this study, almost equal numbers of novel and known miRNAs were detected, suggesting that small RNA-seq analysis is effective in animal species with few known miRNAs, such as horses.

The selection of an appropriate reference miRNA is essential for quantitative miRNA analysis using RT-qPCR [21]. In the quantitative analysis of circulating miRNAs in serum and plasma using RT-qPCR, there is no single universal reference miRNA; it is necessary to select a reference miRNA according to the conditions of each experiment [22]. BestKeeper-1 evaluates the expression stability of a reference gene using threshold cycle (Ct) values obtained by RT-qPCR [23]. In this study, this algorithm was used to evaluate the stability of cel-miR-39, which is a spike-in control used as a reference miRNA in many studies, and miR-191a, let-7g, miR-128, and miR-146a, which were selected from the small RNA-seq results. The miR-146a was selected as a suitable reference miRNA and was found to be more stable than the spike-in control or let-191a and miR-128, which are endogenous miRNAs that are abundant in horse plasma [24]. Therefore, the present study identified a reference miRNA suitable for comparing miRNA variations in horse plasma before and after hydrocortisone administration. The miR-146a was found to be applicable in both the exercise and ACTH administration studies, as there were no differences in the Ct values obtained from either study or the hydrocortisone administration study.

Among the miRNAs that fluctuated significantly after hydrocortisone administration according to small RNA-seq, 20 were selected and validated using RT-qPCR. Nine miRNAs showed significant differences in the RT-qPCR and small RNA-seq results; whereas, the remaining miRNAs showed no significant differences in the RT-qPCR. This difference between the small RNA-seq and RT-qPCR platforms was reported in another study [25], suggesting that RT-qPCR is less likely to yield significant differences. This may be due to differences in reproducibility, sensitivity, specificity, and accuracy between platforms for quantitative miRNA analysis. A previous study reported that the concordance rate of miRNA profiles between small RNA-seq and RT-qPCR was 54.6% [26], which is similar to the concordance rate obtained in this study. Moreover, the difference between RNA-seq and RT-qPCR in this study may have been caused by differences in the amount of RNA used for analysis and reproducibility between the platforms. When miRNAs are applied to drug testing, RT-qPCR is considered more practical because of the cost, time, and amount of RNA required for analysis.

LC-MS/MS, currently used for hydrocortisone drug testing, cannot discriminate between exogenous and endogenous hydrocortisone. Therefore, in this study, the variation during endogenous hydrocortisone elevation was investigated for miRNAs that fluctuated significantly after the administration of hydrocortisone preparations. Endogenous hydrocortisone is secreted from the adrenal cortex upon stimulation with ACTH and is increased by exercise and psychological stress in horses [27,28]. In this study, exercise and ACTH administration were used to create conditions for elevated endogenous hydrocortisone levels in vivo. Both treatments increased the plasma hydrocortisone concentrations, proving that they were able to create the desired state of increased endogenous hydrocortisone. Therefore, this study correctly verified miRNA variations during endogenous hydrocortisone elevation.

The novel miRNAs chr3-33188 and chrX-47614, as well as miR-133a, increased after hydrocortisone administration and decreased or did not change when endogenous hydrocortisone levels increased, suggesting that they may be useful biomarkers for detecting hydrocortisone administration. Additionally, eca-miR-133a is an ortholog of human hsa-miR-133a-3p, which is involved in many diseases, including myocardial disease [29], hepatocellular carcinoma [30], lung cancer [31], and breast cancer [32]. Moreover, miR-133a-3p is involved in drug metabolism [33] and may be involved in the metabolism of hydrocortisone preparations. Orthologs of chr3-33188 and chrX-47614 were not identified in other species.

A limitation of this study is the large difference in plasma hydrocortisone concentrations between hydrocortisone administration and exercise or ACTH treatment. The plasma hydrocortisone concentration increases to approximately 2000 ng/mL an hour after hydrocortisone administration [20]; whereas, the maximum concentration after exercise and ACTH administration is approximately 90 ng/mL, indicating a large discrepancy. This study examined the differences in miRNA variation during exogenous and endogenous hydrocortisone elevation but did not consider the differences in plasma hydrocortisone concentrations between the experiments. Therefore, to more accurately compare miRNA variation during exogenous and endogenous hydrocortisone elevation, it is necessary to investigate a procedure that stimulates endogenous hydrocortisone secretion.

There has been a growing interest in genetic doping, not only in human athletes but also in horse racing. Gene doping involves the introduction of genetic materials that encode proteins in the body for nontherapeutic purposes to improve performance [34]. Currently developed methods for detecting gene doping include the direct detection of introduced genetic material in the blood [35,36]. However, these direct detection methods cannot detect gene doping when exogenous genetic material is incorporated into the nucleus. Indirect detection methods using miRNA biomarkers, such as those identified in this study, may detect biological responses induced by the introduction of genetic material. Thus, an indirect method using miRNAs may be applicable for the detection of gene doping.

## 4. Materials and Methods

### 4.1. Animals and Sample Collection

Six healthy female thoroughbreds (age, 3–10 years old; body weight, 428–530 kg) were used in the hydrocortisone administration study and six healthy thoroughbreds (three geldings and three females; age, 4–8 years old; body weight, 445–600 kg) were used in the exercise and ACTH administration studies. The hydrocortisone administration study was performed on samples from a previously described animal phase [20] in which horses were administered hydrocortisone sodium succinate (Solu-Cortef Injection 100 mg, Pfizer Inc., New York, NY, USA). Because plasma hydrocortisone levels have a circadian rhythm, control samples were collected 17 days prior to hydrocortisone administration and at the same time after the drug administration in each horse as time-matched controls. The horses were housed in individual stalls with ad libitum access to grass, hay, and water. The lights in the stalls were turned off at night and the horses grazed in the paddock during the day without exercise. Experimental procedures were approved by the Animal Welfare and Ethics Committee of the Equine Research Institute, Japan Racing Association (approval numbers: 21-13 and 21-14). The exercise study was performed on a treadmill and each horse completed incremental exercise until exhaustion [37]. Blood samples were collected before exercise and at 1, 5, 10, 20, 40, 60, and 120 min after all-out running. Two months after the exercise experiment, an ACTH administration experiment was performed on the same horses as in the exercise study. Synthetic ACTH formulation (Cortrosyn Injection 0.25 mg, Daiichi Sankyo Co., Tokyo, Japan) was injected intramuscularly into the horses at a dose of 1.0 µg/kg body weight [38,39]. Blood samples were collected at 0.5, 1, 2, 3, 6, and 9 h after administration. Time-matched controls were collected seven days prior to ACTH administration. Blood was collected from the jugular vein into EDTA-containing vacutainers (Japan Becton, Dickinson and Company, Tokyo, Japan) for miRNA analysis and heparin-containing vacutainers (Japan Becton, Dickinson and Company, Tokyo, Japan) for hydrocortisone analysis. Samples were immediately centrifuged at 1500× *g* for 10 min at 4 °C and the plasma samples were transferred and stored at −80 °C prior to analysis.

### 4.2. RNA Extraction and Small RNA-seq

Based on a previous study [20], which showed significant fluctuations in endogenous hydrocortisone levels for up to 48 h post-administration of hydrocortisone preparations, plasma samples within this 48-h time frame were subjected to small RNA-seq. Plasma samples were centrifuged at 17,860× *g* for 30 min at 4 °C prior to RNA extraction to remove cellular debris. Total RNA, including miRNA, was extracted from 1 mL of plasma using the miRNeasy Serum/Plasma Kit (QIAGEN, Hilden, Germany) for small RNA-seq analysis. The quality and quantity of the extracted miRNAs were assessed using a NanoDrop One (Thermo Fisher Scientific, Waltham, MA, USA) and an Agilent TapeStation with RNA Screen Tape (Agilent Technologies, Santa Clara, CA, USA). The NEBNext Small RNA Library Prep Set for Illumina (New England Biolabs, Ipswich, MA, USA) and 1 μg total RNA were used to generate the small RNA-seq library according to the manufacturer’s instructions. Library quality was checked using an Agilent Bioanalyzer with a DNA high-sensitivity chip (Agilent Technologies, Santa Clara, CA, USA). The libraries were sequenced using a HiSeq X system (Illumina, San Diego, CA, USA). Library preparation and sequencing were performed by Macrogen (Tokyo, Japan). Raw reads were deposited in the DNA Data Bank of Japan (DDBJ) under accession number DRA016617.

### 4.3. Bioinformatics Analyses 

Small RNA-seq data were analyzed using the mirseq pipeline (Amelieff Co., Tokyo, Japan). First, quality control was conducted using FastQC version 0.11.9 (Babraham Institute, Cambridge, UK) [40]; then, raw reads were trimmed for sequencing quality and/or adapter contaminations using Cutadapt version 3.4 (TU Dortmund, Nordrhein-Westfalen, Germany) [41] with the following parameters: -b AGATCGGAAGAGCACACGTCTGAACTCCAGTCAC -m 18 -M 30 -q 10. Next, clean reads were aligned against the representative genome of Equus caballus assembly version 3 (EquCab3) and downloaded from Ensembl (https://asia.ensembl.org/Equus_caballus/Info/Index (accessed on 27 July 2023)) using Bowtie version 1.1.1 (Johns Hopkins University, Baltimore, MD, USA) [42] with the following parameters: -*p* 10 -v 0 -k 200. Mapped reads were aligned to the EquCab3 using the mapper function (mapper.pl) in miRDeep2 version 0.1.3 (Centre for Genomic Regulation, Barcelona, Spain) [43] with the following parameters: e -h -i -m -s -t -v -n. The identification of known and novel miRNAs was estimated using the miRDeep2 quantifier function (miRDeep2.pl) with the following parameters: -b 1000. The expression levels of all known and novel miRNAs were estimated using the miRDeep2 quantifier function (quantifier.pl) with default parameters. Differential miRNA levels before and after hydrocortisone administration were analyzed using the DESeq2 statistical software package version 1.34.0 (Fred Hutchinson Cancer Center, Seattle, WA, USA) [44]. Changes in miRNAs were evaluated using the threshold of an adjusted *p*-value (padj) < 0.05. A hierarchical clustering heat map was generated using Heatmapper (University of Alberta, Edmonton, Canada, http://www.heatmapper.ca/ (accessed on 27 July 2023)).

### 4.4. RT-qPCR

The RNA used for RT-qPCR was extracted from 200 μL of plasma, as described above, and 5.6 × 10^8^ copies of the lyophilized *C. elegans* miR-39 miRNA mimic (cel-miR-39, QIAGEN, Hilden, Germany) were added as a spike-in control. Using the Mir-X miRNA First-Strand Synthesis Kit (Takara, Shiga, Japan), 2 μL of miRNA was reverse transcribed into cDNA according to the manufacturer’s instructions. RT-qPCR was performed using the KOD SYBR qPCR Mix (TOYOBO, Osaka, Japan) and a QuantStudio 5 real-time PCR system (Thermo Fisher Scientific, Waltham, MA, USA). The forward primer used was miScript Primer Assay Ce_miR_39_1 from the miRNeasy Serum/Plasma Kit; each miRNA is listed in Supplementary Table S1. The mRQ 3′ primer supplied with the Mir-X miRNA First-Strand Synthesis Kit was used as the reverse primer. The thermal cycling conditions for RT-qPCR included the initial sample incubation at 98 °C for 2 min, followed by 40 cycles of 98 °C for 10 s, 60 °C for 10 s, and 68 °C for 30 s. To quantify the miRNA copy number, the oligonucleotides listed in Supplementary Table S2 containing the corresponding mature miRNA and the adapter sequence of the Mir-X miRNA First-Strand Synthesis Kit were used as standards [7]. Standard oligonucleotides were synthesized using FASMAC (Kanagawa, Japan). The miRNA levels were indicated by dividing the copy number of each target miRNA by the endogenous control miRNA-146a.

### 4.5. Evaluation of Reference miRNAs

To select a reference miRNA for RT-qPCR, the BestKeeper-1 (Technical University of Munich, Munich, Germany) [23] and Ct values obtained by RT-qPCR were used. Five miRNAs were selected as candidate references. These included cel-miR-39 and four endogenous miRNAs, the levels of which were not significantly different before and after hydrocortisone administration; more than 1000 reads were obtained from all samples collected in the hydrocortisone administration experiment using small RNA-seq. The miRNA with the highest correlation coefficient under an SD < 1 was selected as the reference miRNA.

### 4.6. Analysis of Hydrocortisone

The plasma hydrocortisone levels in the samples collected during the exercise and ACTH administration experiments were measured using a LC-MS/MS system, employing a method similar to that used in a previous study [20].

### 4.7. Statistical Analysis

IBM SPSS Statistics 19 (IBM Corp., Armonk, NY, USA) was used for the statistical analysis. The Wilcoxon signed-rank test was performed for the statistical comparison of hydrocortisone and miRNA levels before and after exercise or ACTH administration. Statistical significance was set at *p* < 0.05.

## 5. Conclusions

In this study, the effects of hydrocortisone administration on circulating miRNAs were evaluated; variations in miRNAs that fluctuated significantly with hydrocortisone administration during endogenous hydrocortisone elevation were investigated. As a result, miRNAs that were significantly varied by hydrocortisone administration were identified, including a number of novel miRNAs. The two novel miRNAs and miR-133a showed different variations during exogenous and endogenous hydrocortisone elevation in the plasma. In conclusion, this study provides basic knowledge regarding the plasma miRNA profiles after hydrocortisone administration and suggests that the three miRNAs identified in this study may be useful biomarkers for detecting hydrocortisone administration in doping controls.

## Figures and Tables

**Table 1:** Small RNA-seq data following hydrocortisone administration.

Samples ^1^	Average Raw Reads	Average Clean Reads	Mapped Reads	Number of Novel miRNAs	Number of Known miRNAs
Pre-09	14,709,992	7,270,876	2,645,358	324	319
Pre-10	11,895,794	6,370,296	2,855,883	338	316
Pre-12	12,523,047	6,438,134	2,785,224	334	323
Pre-15	17,089,367	9,692,756	4,379,242	353	316
Pre-18	14,515,643	7,707,896	3,839,293	345	330
Pre-21	16,575,098	9,078,428	4,830,487	353	333
H-1	15,258,351	8,375,329	5,082,109	357	326
H-3	15,244,208	8,685,837	5,436,409	353	330
H-6	15,476,810	8,622,097	4,919,080	347	330
H-9	16,308,131	9,678,295	5,057,211	348	331
H-12	16,497,648	9,086,488	4,410,764	352	318
H-24	14,614,984	8,399,419	4,391,252	342	308
H-36	17,752,135	10,470,181	5,682,381	348	324
H-48	16,278,917	9,159,001	5,045,230	340	330

^1^ Plasma samples were collected before or after hydrocortisone administration and small RNA-seq analysis was performed. The numbers Pre-09 to Pre-21 indicate the time of the day before hydrocortisone administration (pre-administration) as time-matched controls. Numbers H-1 to H-48 represent the time (h) after hydrocortisone administration.

**Table 2:** The stability of five candidate reference miRNAs, as evaluated by BestKeeper-1.

miRNA	cel-miR-39	miR-191a	let-7g	miR-128	miR-146a
SD ^1^	0.506565	0.70237	0.814206	0.718547	0.527002
Coefficient of correlation ^1^	0.852	0.861	0.87	0.899	0.922

^1^ In the BestKeeper-1 algorithm, the miRNA with a standard deviation (SD) of <1 and the highest coefficient of correlation was determined to be the most suitable reference miRNA.

**Table 3:** Differentially expressed circulating miRNAs after hydrocortisone administration.

miRNA ^1^	Time after the Administration (h)	Small RNA-seq		RT-qPCR		
		Level ^2^	log2 Fold-Change	Level ^2^	log2 Fold-Change	*p*-Value
chr3-33188	12	↑	8.66	↑	0.78	0.028
chrX-47614	48	↑	4.19	↑	1.10	0.028
chr20-23348	12	↑	3.48	-	−0.17	0.249
chrX-47606	36	↑	3.34	-	−0.04	0.753
miR-133a	3	↑	2.98	↑	1.25	0.028
miR-206	9	↑	2.75	-	−0.09	0.463
miR-1	3	↑	2.63	↑	1.29	0.046
chr3-32616	6	↑	2.48	-	−0.12	0.249
miR-122	48	↑	2.32	↑	2.88	0.028
chr7-41424	9	↑	2.12	-	0.40	0.116
chr3-33188	3	↓	−11.37	-	0.13	0.345
chr25-30007	12	↓	−4.14	↓	−2.69	0.028
miR-532-3p	9	↓	−2.04	-	0.33	0.116
miR-451	6	↓	−1.93	↓	−1.42	0.028
miR-7	6	↓	−1.80	↓	−0.78	0.046
miR-144	6	↓	−1.69	-	−0.22	0.249
chr7-42879	12	↓	−1.59	↓	−2.90	0.028
miR-142-3p	36	↓	−1.48	-	−0.30	0.075
let-7a	48	↓	−1.47	-	−0.61	0.075
miR-200b	12	↓	−1.46	-	−0.43	0.173

^1^ List of the top 20 miRNAs most altered by hydrocortisone administration in small RNA-seq and their variations by RT-qPCR. The “chr” at the beginning of the name indicates novel miRNAs and the chromosome numbers where they are present. ^2^ Level indicates an increase (↑) or decrease (↓) in miRNA levels after hydrocortisone administration. The “-” indicates no significant difference.

## Data Availability

All data generated by this study are included in the manuscript or in its Supplementary Materials.

## Supplementary Materials

The following information can be downloaded at: https://www.mdpi.com/article/10.3390/ijms241914515/s1.

## References

[1] Bartel D.P. (2004). MicroRNAs: Genomics, biogenesis, mechanism, and function. Cell.

[2] Sohel M.H. (2016). Extracellular/circulating microRNAs: Release mechanisms, functions and challenges. Achiev. Life Sci..

[3] Mitchell P.S., Parkin R.K., Kroh E.M., Fritz B.R., Wyman S.K., Pogosova-Agadjanyan E.L., Peterson A., Noteboom J., O’Briant K.C., Allen A. (2008). Circulating microRNAs as stable blood-based markers for cancer detection. Proc. Natl Acad. Sci. USA.

[4] Saliminejad K., Khorram Khorshid H.R., Soleymani Fard S., Ghaffari S.H. (2019). An overview of microRNAs: Biology, functions, therapeutics, and analysis methods. J. Cell. Physiol..

[5] Ho P.T.B., Clark I.M., Le L.T.T. (2022). MicroRNA-based diagnosis and therapy. Int. J. Mol. Sci..

[6] Lv Y., Lu C., Ji X., Miao Z., Long W., Ding H., Lv M. (2019). Roles of microRNAs in preeclampsia. J. Cell. Physiol..

[7] Ono K., Okamoto S., Ninomiya C., Toji N., Kanazawa T., Ishiguro-Oonuma T., Takahashi T., Iga K., Kizaki K. (2022). Analysis of circulating microRNA during early gestation in Japanese black cattle. Domest. Anim. Endocrinol..

[8] Leuenberger N., Schumacher Y.O., Pradervand S., Sander T., Saugy M., Pottgiesser T. (2013). Circulating microRNAs as biomarkers for detection of autologous blood transfusion. PLoS ONE.

[9] Salamin O., Jaggi L., Baume N., Robinson N., Saugy M., Leuenberger N. (2016). Circulating microRNA-122 as potential biomarker for detection of testosterone abuse. PLoS ONE.

[10] Leuenberger N., Jan N., Pradervand S., Robinson N., Saugy M. (2011). Circulating microRNAs as long-term biomarkers for the detection of erythropoiesis-stimulating agent abuse. Drug Test. Anal..

[11] Kelly B.N., Haverstick D.M., Lee J.K., Thorner M.O., Vance M.L., Xin W., Bruns D.E. (2014). Circulating microRNA as a biomarker of human growth hormone administration to patients. Drug Test. Anal..

[12] Igaz I., Nyírő G., Nagy Z., Butz H., Nagy Z., Perge P., Sahin P., Tóth M., Rácz K., Igaz P. (2015). Analysis of Circulating microRNAs in vivo following Administration of dexamethasone and adrenocorticotropin. Int. J. Endocrinol..

[13] Loup B., André F., Avignon J., Lhuaire M., Delcourt V., Barnabé A., Garcia P., Popot M.A., Bailly-Chouriberry L. (2022). miRNAs detection in equine plasma by quantitative polymerase chain reaction for doping control: Assessment of blood sampling and study of eca-miR-144 as potential erythropoiesis stimulating agent biomarker. Drug Test. Anal..

[14] Blackburn-Munro G., Blackburn-Munro R. (2003). Pain in the brain: Are hormones to blame?. Trends Endocrinol. Metab..

[15] Jankord R., Herman J.P. (2008). Limbic regulation of hypothalamo-pituitary-adrenocortical function during acute and chronic stress. Ann. N. Y. Acad. Sci..

[16] Spiga F., Walker J.J., Terry J.R., Lightman S.L. (2014). HPA axis-rhythms. Compr. Physiol..

[17] Leclere M. (2017). Corticosteroids and immune suppressive therapies in horses. Vet. Clin. N. Am. Equine Pract..

[18] Bohák Z., Szabó F., Beckers J.F., Melo de Sousa N., Kutasi O., Nagy K., Szenci O. (2013). Monitoring the circadian rhythm of serum and salivary cortisol concentrations in the horse. Domest. Anim. Endocrinol..

[19] Larsson M., Edqvist L.E., Ekman L., Persson S. (1979). Plasma cortisol in the horse, diurnal rhythm and effects of exogenous ACTH. Acta Vet. Scand..

[20] Kikuchi M., Nagata S., Ishige T., Minamijima Y., Hirota K., Tozaki T., Kakoi H., Kizaki K. (2023). Evaluation of the effect of glucocorticoid treatment on adrenocortical functions by monitoring endogenous hydrocortisone in horses. J. Vet. Med. Sci..

[21] Lardizábal M.N., Nocito A.L., Daniele S.M., Ornella L.A., Palatnik J.F., Veggi L.M. (2012). Reference genes for real-time PCR quantification of microRNAs and messenger RNAs in rat models of hepatotoxicity. PLoS ONE.

[22] Pagacz K., Kucharski P., Smyczynska U., Grabia S., Chowdhury D., Fendler W. (2020). A systemic approach to screening high-throughput RT-qPCR data for a suitable set of reference circulating miRNAs. BMC Genom..

[23] Pfaffl M.W., Tichopad A., Prgomet C., Neuvians T.P. (2004). Determination of stable housekeeping genes, differentially regulated target genes and sample integrity: BestKeeper—Excel-based tool using pair-wise correlations. Biotechnol. Lett..

[24] Lee S., Hwang S., Yu H.J., Oh D., Choi Y.J., Kim M.C., Kim Y., Ryu D.Y. (2016). Expression of microRNAs in horse plasma and their characteristic nucleotide composition. PLoS ONE.

[25] Tiedt S., Prestel M., Malik R., Schieferdecker N., Duering M., Kautzky V., Stoycheva I., Böck J., Northoff B.H., Klein M. (2017). RNA-seq identifies circulating miR-125a-5p, miR-125b-5p, and miR-143-3p as potential biomarkers for acute ischemic stroke. Circ. Res..

[26] Mestdagh P., Hartmann N., Baeriswyl L., Andreasen D., Bernard N., Chen C., Cheo D., D’Andrade P., DeMayo M., Dennis L. (2014). Evaluation of quantitative miRNA expression platforms in the microRNA quality control (miRQC) study. Nat. Methods.

[27] Foreman J.H., Ferlazzo A. (1996). Physiological responses to stress in the horse. Pferdeheilkunde Equine Med..

[28] Schmidt A., Biau S., Möstl E., Becker-Birck M., Morillon B., Aurich J., Faure J.M., Aurich C. (2010). Changes in cortisol release and heart rate variability in sport horses during long-distance road transport. Domest. Anim. Endocrinol..

[29] Xiao Y., Zhao J., Tuazon J.P., Borlongan C.V., Yu G. (2019). MicroRNA-133a and myocardial infarction. Cell Transplant..

[30] Liang H.W., Yang X., Wen D.Y., Gao L., Zhang X.Y., Ye Z.H., Luo J., Li Z.Y., He Y., Pang Y.Y. (2018). Utility of miR-133a-3p as a diagnostic indicator for hepatocellular carcinoma: An investigation combined with GEO, TCGA, meta-analysis and bioinformatics. Mol. Med. Rep..

[31] Xu M., Wang Y.Z. (2013). miR-133a suppresses cell proliferation, migration and invasion in human lung cancer by targeting MMP-14. Oncol. Rep..

[32] Li Z., Xu W., Ren X., Xu J., Chen J. (2019). Puerarin promotes DUSP1 expression by regulating miR-133a-3p in breast cancer. Mol. Med. Rep..

[33] Yu A.M., Tian Y., Tu M.J., Ho P.Y., Jilek J.L. (2016). MicroRNA pharmacoepigenetics: Posttranscriptional regulation mechanisms behind variable drug disposition and strategy to develop more effective therapy. Drug Metab. Dispos..

[34] Gould D. (2013). Gene doping: Gene delivery for Olympic victory. Br. J. Clin. Pharmacol..

[35] Sugasawa T., Nakano T., Fujita S.I., Matsumoto Y., Ishihara G., Aoki K., Yanazawa K., Ono S., Tamai S., Manevich L. (2021). Proof of gene doping in a mouse model with a human erythropoietin gene transferred using an adenoviral vector. Genes.

[36] Tozaki T., Ohnuma A., Kikuchi M., Ishige T., Kakoi H., Hirota K., Kusano K., Nagata S. (2020). Microfluidic quantitative PCR detection of 12 transgenes from horse plasma for gene doping control. Genes.

[37] Nagata S., Takeda F., Kurosawa M., Mima K., Hiraga A., Kai M., Taya K. (1999). Plasma adrenocorticotropin, cortisol and catecholamines response to various exercises. Equine Vet. J. Suppl..

[38] Caloni F., Spotti M., Villa R., Mariani C., Montana M., Pompa G. (1999). Hydrocortisone levels in the urine and blood of horses treated with ACTH. Equine Vet. J..

[39] Kirchmeier A., van Herwaarden A.E., van der Kolk J.H., Sauer F.J., Gerber V. (2020). Plasma steroid profiles before and after ACTH stimulation test in healthy horses. Domest. Anim. Endocrinol..

[40] Babraham Bioinformatics—FastQC A Quality Control Tool for High Throughput Sequence Data. https://www.bioinformatics.babraham.ac.uk/projects/fastqc.

[41] Martin M. (2011). Cutadapt removes adapter sequences from high-throughput sequencing reads. EMBnet J..

[42] Langmead B., Trapnell C., Pop M., Salzberg S.L. (2009). Ultrafast and memory-efficient alignment of short DNA sequences to the human genome. Genome Biol..

[43] Friedländer M.R., Mackowiak S.D., Li N., Chen W., Rajewsky N. (2012). miRDeep2 accurately identifies known and hundreds of novel microRNA genes in seven animal clades. Nucleic Acids Res..

[44] Love M.I., Huber W., Anders S. (2014). Moderated estimation of fold change and dispersion for RNA-seq data with DESeq2. Genome Biol..

